# Investigating the capability of deep learning models to predict age and biological sex from anterior segment ophthalmic imaging: a multi-centre retrospective study

**DOI:** 10.1136/bmjopen-2025-107196

**Published:** 2025-10-29

**Authors:** Shafi Balal, Laurence Cox, Ajmal Khan, Lynn Kandakji, Marcello Leucci, Pearse A Keane, Daniel Gore, Nikolas Pontikos, Bruce Allan

**Affiliations:** 1 University College London Institute of Ophthalmology, London, UK; 2 Moorfields Eye Hospital NHS Foundation Trust, London, UK; 3 Anglia Ruskin University, Chelmsford, UK; 4 University College London, London, UK

**Keywords:** OPHTHALMOLOGY, Corneal and external diseases, Machine Learning

## Abstract

**Abstract:**

**Objective:**

To assess the capability of a convolutional neural network trained by transfer learning on anterior segment optical coherence tomography (AS-OCT) images, Placido-disk corneal topography images and external photographs to predict age and biological sex.

**Design:**

Development of a deep learning model trained on retrospectively collected data using transfer learning.

**Setting:**

A multicentre secondary care public health trust based in London.

**Participants:**

We included 557,468 scans from 40,592 eyes of 20,542 patients. Data were extracted from all patients who underwent MS-39 imaging within our trust from October 2020 to March 2023.

**Primary and secondary outcome measures:**

Primary outcome measures for biological sex classification included accuracy, precision, recall, F1-score and area under the receiver operating curve (ROC-AUC). Primary outcome measures for age prediction were Pearson correlation coefficients (r), coefficients of determination (R²) and the mean absolute error (MAE) to evaluate the predictive performance. The secondary outcome was to visualise and interpret the model’s decision-making process through the construction of saliency maps.

**Results:**

For age prediction, the MAEs for the Placido, AS-OCT and external photograph models were 5.2, 5.1 and 6.2 years, respectively. For gender classification, the same models achieved ROC-AUCs of 0.88, 0.73 and 0.81, respectively. No difference in performance was found in the analysis of corneas with pathological topography. The saliency maps highlighted the peri-limbal cornea for age prediction and the central cornea for gender discrimination.

**Conclusions:**

Our study demonstrates that deep learning models can extract age and gender information from anterior segment images. These findings support the concept that the anterior segment, like the retina, encodes important biological information. Future research should explore whether these models can predict specific systemic conditions.

**STRENGTHS AND LIMITATIONS OF THIS STUDY**

- As the study was conducted within a large multi-ethnic multi-centre public health trust and included images across a broad spectrum of corneal diseases as well as images of healthy corneas, the sample population was highly heterogenous.
- The large sample size ensured adequate training of deep learning models.
- The use of gradient-weighted class activation mapping and other saliency mapping techniques to visualise the models’ decision-making process.
- The potential selection bias from a hospital-based population with a greater representation of corneal pathology compared with that within the general population.

## Introduction

 The utility of ocular analysis in the evaluation of systemic health status, particularly cardiovascular health, is well established.^1^ However, it is increasingly being realised that ocular imaging analyses may have predictive capabilities extending well beyond what could previously have been imagined. Using deep learning techniques, it has recently been demonstrated that both age and gender can be predicted from fundus photographs and macula optical coherence tomography (OCT).^2^,^5^ For example, Poplin *et al*^6^ demonstrated that their deep learning algorithm could differentiate subject sex from colour fundus photographs with 97% accuracy. Predicting age and biological sex represents a paradigm shift in ocular assessment that was previously inconceivable even to clinicians who dedicated their careers to examining retinas.

Biological age gap metrics (derived from the difference between chronological age and biological age predictions from deep learning analyses) have demonstrated theoretical value for predicting disease progression and mortality risk, as evidenced in studies examining the brain age gap.^7^ Building on these findings, Zhu *et al*^8^ established that deep learning models applied to retinal images can effectively predict biological age, with the resulting retinal age gap serving as a minimally invasive and potentially useful supplementary tool for cardiovascular risk stratification. This is an example of the rapidly developing field of oculomics,^9^ which treats the retina as a privileged window for non-invasive studies of systemic health status. If similar biological signals are encoded in the anterior segment (cornea, anterior chamber and iris) of the eye, it should be possible to develop predictive models for systemic health based on anterior segment imaging. This predictive potential has previously been suggested by the work of Babenko *et al,* which demonstrated that external ocular photographs can be used to predict not only diabetic retinopathy and diabetic macular oedema but also measures of systemic health, ranging from HbA1c to markers of both liver and renal function.^10 11^

Previous anterior segment OCT (AS-OCT) studies have suggested that biomarkers for age and gender are present. These include age-related increases in iris width with significantly greater dimensions in males.^12^ However, unlike the extensive body of work in this field for the retina,^3 5 6 13 14^ studies examining the predictive capabilities of models derived from anterior segment imaging remain limited.^15^

This study aimed to demonstrate that deep learning could be used to analyse anterior segment ophthalmic images, specifically spectral domain AS-OCT, external eye photographs and Placido disc corneal topography, to classify biological sex and to predict patient age in a multi-ethnic dataset. We also aimed to localise anatomical regions within the images contributing most to the predictive power of the new models we developed.

## Methods

### Study setting and design

An anonymised multi-centre data extract was obtained from all consecutive patients who had MS-39 scans at Moorfields Eye Hospital and its peripheral units between October 2020 and March 2023 within London and the surrounding regions. All AS-OCT images included in the analyses were from patients ≥16 years of age. Images from patients with a documented history of keratoplasty were excluded. We retrieved baseline demographic data (age, gender and ethnicity) using anonymised extracts from the electronic healthcare record system included in the research database. Patient level split was performed to avoid data leakage. This study adhered to the tenets of the Declaration of Helsinki. This study involves human participants but was not approved by an Ethics Committee or Institutional Board. As an observational study of anonymised healthcare data, under article 9 exemptions in UK-GDPR, no informed consent or ethical review was required. The dataset was accessed through the INSIGHT^16^ virtual research environment (HRA ref: 20/WS/0087). Patients or the public were not involved in the design, conduct, reporting, or dissemination plans of our research.

### Instrumentation

All data were obtained using the MS-39 AS-OCT (CSO Italia, Florence, Italy), which employs a combination of spectral domain-OCT (SD-OCT) and Placido-disk corneal topography to image the anterior segment of the eye. In the default acquisition mode, the MS-39 captures a single Placido top-view image and a set of 25 radial SD-OCT scans. The manufacturer’s instructions were followed by all operators, and all measurements were made in mesopic luminance. Scans flagged as poor quality by the manufacturers' acquisition software were discarded at the time of data acquisition. Images which had poor sectional coverage (Placido < 65% or OCT < 85%) were excluded. The MS-39 automated corneal classification algorithm was used to label scans as normal, abnormal and keratoconus. We classed ‘abnormal’ as any cornea, which was classified by the MS-39’s internal algorithm as ‘abnormal’ with a probability score of >0.5. This likely referred to eyes which had significant corneal scarring or an ectasia other than keratoconus.

### Data preprocessing

Three types of grayscale images were included: Placido, AS-OCT and external eye images. The preprocessing pipeline included normalisation to the range [0,1], resizing to 224×224 pixels using bilinear interpolation and conversion to PyTorch tensors. For the OCT images, we resized each image to 224×224 pixels using bilinear interpolation and normalised each image individually based on its minimum and maximum pixel values to enhance local contrast and structural details. Data augmentation techniques, including random rotation (±20°) and horizontal flipping, were applied to enhance model generalisation.

### Model development

**Figure 1:**
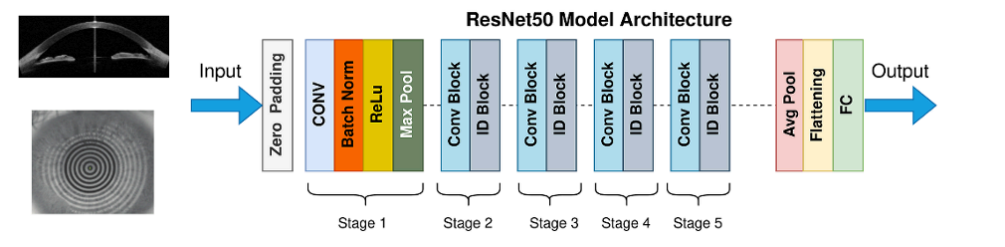
Schematic of the pre-trained ResNet-50 architecture in which the model received either anterior segment OCT, external photo or Placido image inputs. Following a Zero-Padding layer, the model consists of five main convolutional stages before the output was generated by the Average Pooling, Flattening and Fully Connected Layers. ReLU, Rectified Linear Unit; OCT, optical coherence tomography; FC, Fully Connected.

Separate deep learning models were developed for biological sex classification and for age estimation. Both models were implemented, trained and evaluated in PyTorch, leveraging 2 x Nvidia T4 graphic processing units (GPUs). We implemented and compared several deep learning model architectures, including a custom CNN, ResNet-50^17^ and Vision Transformer (ViT-B/16).^18^ The ResNet-50 model showed the best performance and was selected as our final architecture (figure 1).

For gender classification, we implemented a transfer learning approach using a pre-trained ResNet-50 architecture (figure 1). The model was initialised with weights pre-trained on ImageNet and modified by replacing the final fully connected layer with a custom classification head consisting of a 256-neuron hidden layer followed by binary sigmoid output for gender prediction. We employed a progressive training strategy, where initially only the custom classification layers were trained while keeping the convolutional backbone frozen, followed by selective unfreezing of deeper network layers after five epochs to enable fine-tuning with a reduced learning rate (0.0001). The Adam optimizer with a weight decay of 1e-4 was employed for training, using binary cross-entropy as the loss function. The initial learning rate was set to 0.001, with a reduction factor of 0.1 when validation loss plateaued. The dataset was divided into training (85%) and testing (15%) sets using stratified sampling to maintain gender distribution. Model development used fivefold cross-validation on the (85%) training portion, while the hold-out (15%) test set was reserved exclusively for final model evaluation. The algorithm was trained for 50 epochs and incorporated early stopping with a patience of five epochs to prevent overfitting.

The age estimation model shared the same fundamental CNN architecture as the gender classification model, with the output layer modified to produce a continuous value rather than a binary classification. The model was trained using mean squared error (MSE) as the loss function to minimise age prediction errors. Performance was evaluated using both MSE and MAE in years.

### Statistical analysis

For binary classification (biological sex), evaluation included accuracy, precision, recall, F1-score and area under the receiver operating curve (ROC-AUC) with 95% CIs to assess model performance. CIs were calculated using the bootstrapping method (2000 iterations). Subgroup analysis compared AUCs of normal versus abnormal corneas using DeLong’s test.

For the continuous variable (age prediction), we reported Pearson correlation coefficients (r), coefficients of determination (R²), and the mean absolute error (MAE) to evaluate the predictive performance. One-way ANOVA was used to test the difference between the absolute errors.

The data were analysed using R 4.5.0 (R Foundation for Statistical Computing) and Python 3.12 (Python Software Foundation, Wilmington, DE). In the analyses, p<0.05 was considered statistically significant.

### Visual explanation of model decisions

To visualise and interpret the model’s decision-making process, we employed Gradient-weighted Class Activation Mapping (Grad-CAM)^19^ on the trained ResNet-50 model. We extracted averaged activation maps from 50 patients of a similar age and the same gender from the last convolutional layer and weighted these feature maps by the gradients of the output with respect to the feature maps.

## Results

### Dataset characteristics

We included 557 468 scans from 40 592 eyes of 20 542 patients of whom 39.3% had keratoconus, 25.2% were normal and 35.5% abnormal. The mean age of this cohort was 46.7±20.1 years (range 16–94), with males accounting for 55.9% of the sample. The ethnicity distribution from the 54% of available data was: 37.22% White, 25.87% Asian, 24.04% other ethnicities and 12.87% Black.

### Age prediction

The mean correlation coefficients, R² and MAE for external photo, Placido and AS-OCT models are shown in table 1.

**Table 1:** Tabulated performance of the Placido, external photo and AS-OCT models for both age prediction and gender classification

	Age Prediction					
	Fivefold Cross Validation			Test Set		
	Placido	AS-OCT	External Photo	Placido	AS-OCT	External Photo
Mean Correlation Coefficient	0.92	0.92	0.88	0.94 (0.93–0.95)	0.91 (0.89–0.93)	0.87 (0.85–0.90)
R²	0.85 (0.84–0.86)	0.86 (0.85–0.87)	0.81 (0.79–0.83)	0.87 (0.85–0.89)	0.89 (0.87–0.91)	0.78 (0.75–0.80)
Mean Absolute Error (Years)	5.3 (5.1–5.5)	5.2 (5.0–5.4)	6.4 (6.2–6.6)	5.2 (4.9–5.5)	5.1 (4.9–5.3)	6.2 (5.9–6.5)

	Biological Sex Classification					
	Five-Fold Cross Validation			Test Set		
	Placido	AS-OCT	External Photo	Placido	AS-OCT	External Photo
Accuracy	77.11% (73.5–80.7)	67.35% (63.8–70.9)	71.35% (68.2–74.5)	79.92% (78.1–81.7)	65.79% (63.8–67.8)	73.42% (70.8–77.8)
ROC-AUC	0.86 (0.83–0.89)	0.75 (0.71–0.79)	0.78 (0.74–0.82)	0.88 (0.86–0.90)	0.73 (0.71–0.75)	0.81 (0.78–0.84)
Precision	0.74 (0.70–0.78)	0.72 (0.68–0.76)	0.72 (0.68–0.76)	0.80 (0.78–0.82)	0.70 (0.68–0.72)	0.75 (0.72–0.77)
Recall	0.76 (0.72–0.80)	0.39 (0.34–0.44)	0.59 (0.54–0.64)	0.75 (0.72–0.77)	0.37 (0.35–0.39)	0.62 (0.60–0.64)

Both the average from the fivefold cross validation and test data sets are shown. Parentheses contain the range for averages from cross validation and confidence intervals for test set, respectively.

AS-OCT, anterior segment optical coherence tomography.

**Figure 2:**
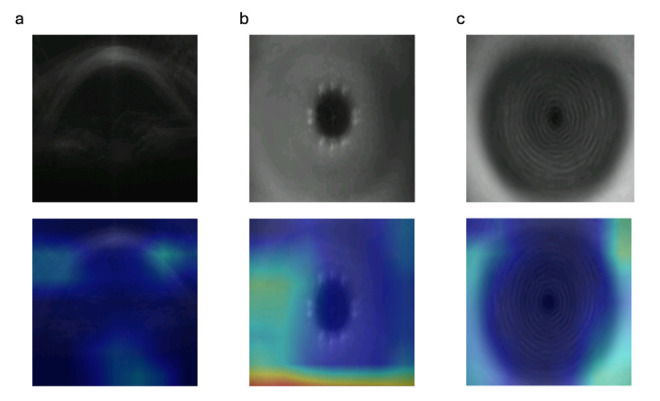
Averaged GradCAM from 50 patients showing areas of the images in which discriminating features for age prediction are concentrated. The heat map (blue to red) indicates areas of highest importance, with brighter regions showing the strongest contribution for AS-OCT (**a**), external photos (**b**) and Placido (**c**). AS-OCT, anterior segment optical coherence tomography.

**Figure 3:**
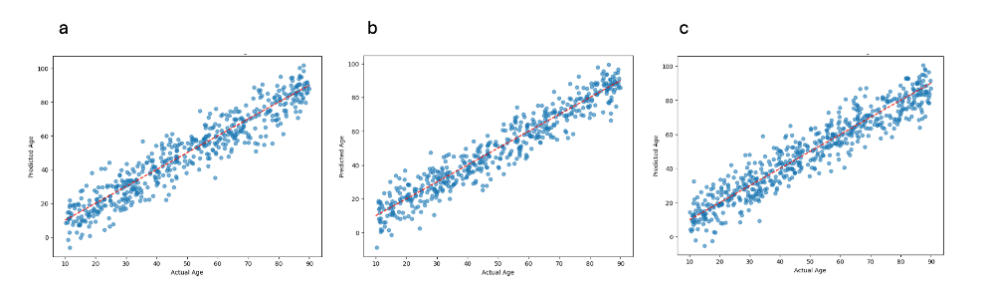
Relationship between actual and predicted age for external photograph (**a**) AS-OCT (**b**), and Placido (**c**) models. AS-OCT, anterior segment optical coherence tomography

For all three models, the saliency maps highlighted the peri-limbal cornea as the most significant part of the image in predicting age, as shown in figure 2. The AS-OCT saliency map also highlighted the lens, as shown in figure 2a. Figure 3 shows the predictions of each model closely tracked actual age across the entire age spectrum from adolescence to elderly subjects.

### Biological sex prediction

**Figure 4:**
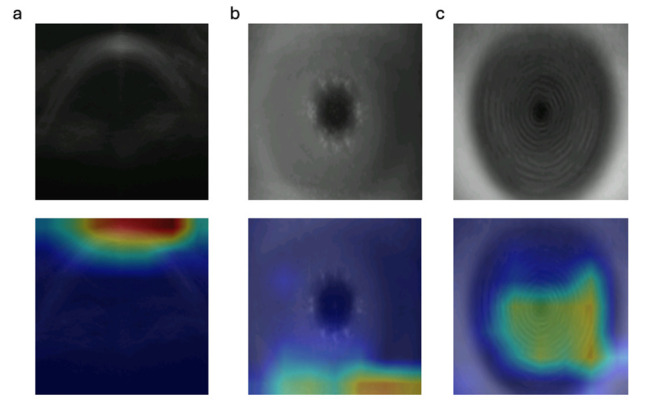
Averaged GradCAM visualisation showing areas of the images in which discriminating features for biological sex prediction are concentrated. The heat map (blue to red) indicates areas of highest importance, with brighter regions showing the strongest contribution for AS-OCT (**a**), external photos (**b**) and Placido (**c**). AS-OCT, anterior segment optical coherence tomography.

The evaluation metrics are displayed in table 1. The Placido model showed the highest AUC (0.88), followed by external photos (0.81) and AS-OCT (0.73). Figure 4 shows that for the AS-OCT (figure 4a) and Placido (figure 4c) models, the central cornea contributed most significantly to the function’s output, while for the external photo (figure 4b) trained model, the inferior perilimbal cornea contributed most significantly.

### Subgroup analysis

Subgroup analysis was conducted to evaluate model performance across normal, keratoconus and abnormal corneas. For age prediction, MAE was comparable across normal (5.3 years), keratoconus (5.3 years) and other pathologies (5.4 years), with no statistically significant differences between groups (p=0.28). Similarly, gender prediction showed consistent performance with ROC-AUC values of 0.74, 0.72,and 0.73 for normal, keratoconus and abnormal groups, respectively (p=0.42). Model performance was also evaluated across different ethnic groups. For age prediction, MAE revealed no statistically significant differences (p=0.39). For gender prediction, ROC-AUC values were compared across the same ethnic subgroups, also showing no statistically significant differences (p=0.26).

## Discussion

Our study demonstrates that deep learning models can predict both age and gender from anterior segment images with good accuracy. Specifically, our models achieved encouraging performance in age prediction from Placido disc corneal topography (MAE=5.2 years), AS-OCT scans (MAE=5.1 years) and external photographs (MAE=6.2 years). Gender prediction performance was notably stronger with Placido images (AUC 0.88) compared with both external photos (AUC 0.81) and AS-OCT images (AUC 0.73).

Our findings align with and extend the work of Lee *et al*,^15^ who demonstrated that their vision transformer had excellent categorical discrimination between older and younger subjects (AUC 0.91 for ≤75 vs >75 years) for AS-OCT images. Our regression-based approach offers continuous age estimation across the entire age spectrum. Notably, like Lee *et al*, our study also identified the peripheral cornea and iridocorneal angle region as key contributors to age predictions based on saliency maps. However, while Lee *et al* found modest performance for gender prediction from AS-OCT (AUC 0.66), our study demonstrated substantially better gender classification from Placido images (AUC 0.88), AS-OCT (AUC 0.73) and external photographs (AUC 0.81). This difference could be explained by our larger dataset with 278 734 AS-OCT images compared with their 2970. In their retinal age gap study, Zhu *et al*^8^ showed that the difference between predicted biological age and chronological age could serve as a biomarker for mortality risk. The capability of models trained on anterior segment images for this purpose requires further investigation.

Age prediction and biological sex classification accuracy for deep learning models derived from anterior segment imaging presented here approaches that previously demonstrated for models derived from retinal imaging. Age prediction accuracy for retinal imaging is between 3.3 and 5.8 years,^13 20^ only marginally superior performance to the MAE of 5.1 years for our best performing model. AUCs for prediction of gender from retinal imaging are between 0.8 and 0.96.^20^ In comparison, only our AUCs for models derived from Placido images (AUC 0.88) and external photographs (AUC 0.81) were within this range. The superior accuracy obtained by models derived from Placido and external photography over AS-OCT images may be explained by the presence of anatomical structures such as the upper eyelid and the limbal vasculature in these images. Nonetheless, there is clearly some utility of both Placido-based imaging and external photography beyond any features in these regions, given the significance of the inferior and central cornea in the aggregated feature maps (figure 4b,c). The AUC of 0.73 for gender prediction with AS-OCT indicates a moderate discriminative ability, which is likely due to the known gender differences in corneal anatomy such as a greater tendency for a steeper cornea and higher astigmatism in female eyes.^21^ There is also evidence to suggest that women have narrower iridocorneal angles,^22 23^ though this was not highlighted in our saliency map (figure 4a).

More recently, it has become plausible that, through deep learning, fundus imaging could represent a useful supplementary indicator for a variety of systemic health conditions including neurodegenerative conditions such as Alzheimer’s^24 25^ or Parkinson’s disease,^14^ as well as cardiovascular diseases.^26 27^ This field, termed ‘oculomics’, may extend to anterior segment imaging with distinct biomarkers for systemic disease. Findings from this study have demonstrated that hidden biological signatures are present within the anterior segment ocular anatomy. The AlzEye and UK biobank datasets are proving essential to the development of retinal image-based oculomics, and further investigation into the association between anterior segment features and systemic health will require the inclusion of anterior segment imaging in similar biobank studies or wider ranging observational healthcare datasets.^28 29^

The results for AS-OCT- and Placido-trained CNNs for predicting age were comparable, and the external photograph model was marginally inferior. While all three models primarily detected changes in the perilimbal cornea (figure 2), the AS-OCT model also detected changes in the central cornea as well as the lens. These findings from saliency maps seem highly plausible given that previous research has demonstrated that senile corneal thinning occurs primarily towards the periphery.^30^ The development of arcus is also strongly associated with increasing age, and central corneal thickness is also known to decrease throughout life.^31^,^33^ Furthermore, it is unsurprising that the AS-OCT saliency map highlights the lens due to the higher probability of either cataracts or pseudophakia in older individuals.

The strengths of our study include the use of a highly heterogenous multi-ethnic sample across a spectrum of corneal conditions with a large sample size that facilitated effective model training. The use of Grad-CAM and other saliency mapping techniques allowed us to understand how age and gender information is concentrated in anterior segment images, moving beyond a ‘black box’ approach. The main limitation of this study is the potential selection bias from a hospital-based population. Additionally, while our models performed well across ethnicities represented in our dataset, external validation from other populations would strengthen the generalisability of our findings.

## Conclusions

In conclusion, our study demonstrates that deep learning models can extract age and gender information from anterior segment images. These findings support the concept that the anterior segment, like the retina, encodes important biological information and may serve as an accessible window for assessing both ocular and systemic health parameters. Future research should explore whether these models can be extended to predict specific systemic conditions.

## Data Availability

Data may be obtained from a third party and are not publicly available.

## References

[1] Frith E, Loprinzi PD (2018). Retinopathy and Mortality. *Diabetes Spectr*.

[2] Chen R, Zhang S, Peng G (2024). Deep neural network-estimated age using optical coherence tomography predicts mortality. Geroscience.

[3] Chueh K-M, Hsieh Y-T, Huang S-L (2020). Prediction of gender from macular optical coherence tomography using deep learning. Invest Ophthalmol Vis Sci.

[4] Korot E, Pontikos N, Liu X (2021). Predicting sex from retinal fundus photographs using automated deep learning. Sci Rep.

[5] Wang Y, Liu T, Williamson D (2023). Age prediction from retinal fundus images and segmented vessel images using deep learning. Invest Ophthalmol Vis Sci.

[6] Poplin R, Varadarajan AV, Blumer K (2018). Prediction of cardiovascular risk factors from retinal fundus photographs via deep learning. Nat Biomed Eng.

[7] Tanveer M, Ganaie MA, Beheshti I (2023). Deep learning for brain age estimation: A systematic review. *Information Fusion*.

[8] Zhu Z, Shi D, Guankai P (2023). Retinal age gap as a predictive biomarker for mortality risk. Br J Ophthalmol.

[9] Wu J-H, Liu TYA (2022). Application of Deep Learning to Retinal-Image-Based Oculomics for Evaluation of Systemic Health: A Review. J Clin Med.

[10] Babenko B, Traynis I, Chen C (2023). A deep learning model for novel systemic biomarkers in photographs of the external eye: a retrospective study. Lancet Digit Health.

[11] Babenko B, Mitani A, Traynis I (2022). Detection of signs of disease in external photographs of the eyes via deep learning. Nat Biomed Eng.

[12] Invernizzi A, Giardini P, Cigada M (2015). Three-Dimensional Morphometric Analysis of the Iris by Swept-Source Anterior Segment Optical Coherence Tomography in a Caucasian Population. Invest Ophthalmol Vis Sci.

[13] Shigueoka LS, Mariottoni EB, Thompson AC (2021). Predicting Age From Optical Coherence Tomography Scans With Deep Learning. Trans Vis Sci Tech.

[14] Tran C, Shen K, Liu K (2024). Deep learning predicts prevalent and incident Parkinson’s disease from UK Biobank fundus imaging. Sci Rep.

[15] Lee YJ, Choe S, Wy S (2022). Demographics Prediction and Heatmap Generation From OCT Images of Anterior Segment of the Eye: A Vision Transformer Model Study. Trans Vis Sci Tech.

[16] Bilton EJ, Guggenheim EJ, Baranyi B (2023). A Datasheet for the INSIGHT University Hospitals Birmingham Retinal Vein Occlusion Data Set. *Ophthalmol Sci*.

[17] He K, Zhang X, Ren S (2016). Deep residual learning for image recognition.

[18] Dosovitskiy A, Beyer L, Kolesnikov A (2020). An image is worth 16x16 words: Transformers for image recognition at scale. arXiv.

[19] Selvaraju RR, Cogswell M, Das A (2017). Grad-cam: visual explanations from deep networks via gradient-based localization.

[20] Rim TH, Lee G, Kim Y (2020). Prediction of systemic biomarkers from retinal photographs: development and validation of deep-learning algorithms. Lancet Digit Health.

[21] De Bernardo M, Zeppa L, Zeppa L (2020). Biometric Parameters and Corneal Astigmatism: Differences Between Male and Female Eyes. *Clin Ophthalmol*.

[22] Fernández-Vigo JI, García-Feijóo J, Martínez-de-la-Casa JM (2016). Fourier domain optical coherence tomography to assess the iridocorneal angle and correlation study in a large Caucasian population. BMC Ophthalmol.

[23] Rizk M, Grise-Dulac A, Gatinel D (2024). Glaucoma in women: What do we know so far - A systematic review. *AJO International*.

[24] Cheung CY, Ran AR, Wang S (2022). A deep learning model for detection of Alzheimer’s disease based on retinal photographs: a retrospective, multicentre case-control study. Lancet Digit Health.

[25] Wisely CE, Wang D, Henao R (2022). Convolutional neural network to identify symptomatic Alzheimer’s disease using multimodal retinal imaging. Br J Ophthalmol.

[26] Gargeya R, Leng T (2017). Automated Identification of Diabetic Retinopathy Using Deep Learning. Ophthalmology.

[27] Zhang L, Yuan M, An Z (2020). Prediction of hypertension, hyperglycemia and dyslipidemia from retinal fundus photographs via deep learning: A cross-sectional study of chronic diseases in central China. PLoS ONE.

[28] Warwick AN, Curran K, Hamill B (2023). UK Biobank retinal imaging grading: methodology, baseline characteristics and findings for common ocular diseases. *Eye (Lond*).

[29] Wagner SK, Hughes F, Cortina-Borja M (2022). AlzEye: longitudinal record-level linkage of ophthalmic imaging and hospital admissions of 353 157 patients in London, UK. BMJ Open.

[30] Ma R, Liu Y, Zhang L (2021). Changes in Corneal Morphology with Age in Asian Population: A Multicenter Study of 30,618 Cases. Adv Ther.

[31] Galgauskas S, Juodkaite G, Tutkuvienė J (2014). Age-related changes in central corneal thickness in normal eyes among the adult Lithuanian population. Clin Interv Aging.

[32] Hashemi H, Malekifar P, Aghamirsalim M (2022). Prevalence and associated factors of corneal arcus in the geriatric population; Tehran geriatric eye study. BMC Ophthalmol.

[33] Vurgese S, Panda-Jonas S, Saini N (2011). Corneal Arcus and Its Associations with Ocular and General Parameters: The Central India Eye and Medical Study. Invest Ophthalmol Vis Sci.

