# Supplementary material for: Investigating the capability of deep learning models to predict age and biological sex from anterior segment ophthalmic imaging: a multi-centre retrospective study
Source: BMJ Open. 2025 Oct 29;15(10):e107196. doi: 10.1136/bmjopen-2025-107196 (PMC12574434; doi:10.1136/bmjopen-2025-107196)
Supplement: Supplementary file 1 [file bmjopen-2025-107196.reviewer_comments.pdf]

PEER REVIEW HISTORY

BMJ Open publishes all reviews undertaken for accepted manuscripts. Reviewers are asked to complete a checklist review form and are provided with free text boxes to elaborate on their assessment. These free text comments are reproduced below.

ARTICLE DETAILS

Title (Provisional)

Investigating the capability of deep learning models to predict age and biological sex from anterior segment ophthalmic imaging: a multi-centre retrospective study

Authors

Balal, Shafi; Cox, Laurence; Khan, Ajmal; Kandakji, Lynn; Leucci, Marcello; Keane, Pearse A; Gore, Daniel; Pontikos, Nikolas; Allan, Bruce

---

VERSION 1 - REVIEW

**Reviewer**	**1**
**Name**	**Zeboulon, Pierre**
**Affiliation**	**Hôpital Fondation Adolphe de Rothschild**
**Date**	**01-Aug-2025**
**COI**	**None**

I read with interest this manuscript. I would like to congratulate the authors for the quality of the study and manuscript.

I have minor comments :

-it is mentioned that the aspect ratio of OCT images is preserved. As the images are not squared and they were rescaled to 224,224 I would like to understand what you did

- sampling is mentioned to be stratified on gender, but was care taken to make sure both eyes (and all images) of a given patient are either in the training or test ? In other words, has sampling been performed at the patient level?

Finally regarding figure 2 and 4, it might be interesting to average results from the same sex and similar ages instead of random samples. Was this performed ?

great job otherwise !

**Reviewer**	**2**
**Name**	**Yamaguchi, Takefumi**
**Affiliation**	**Tokyo Dental College**

**Date**	**09-Aug-2025**
**COI**	**None**

This is an interesting study, reporting a novel DL model to predict biological sex and age using AS-OCT images. The manuscript is well-written and the study sounds fair.

Minor comments

1. I wonder if there were prediction error differences in ethnicity, age groups (younger than 30, or over 60) and disease (KCN, normal, and other disease) in the prediction, ROC-AUC, accuracy, and other performance indices.

2. The authors included 557468 scans from 40592 eyes. Please describe how the authors selected the images.

3. Please clarify "abnormal" eyes in detail.

4. I wonder if the accuracy of Placido exceeded AS-OCT or photos, because the tear film. Please address the comorbidity of dry eye disease.

5. Page 15, Line 25: "There was also a clear trend towards less accurate age prediction for older subjects (Fig 3)." Please show this using some statistical analysis.

VERSION 1 - AUTHOR RESPONSE

Reviewer: 1
Dr. Pierre Zeboulon, Hôpital Fondation Adolphe de Rothschild

Comments to the Author:
I read with interest this manuscript. I would like to congratulate the authors for the quality of the study and manuscript. I have minor comments :

-it is mentioned that the aspect ratio of OCT images is preserved. As the images are not squared and they were rescaled to 224,224 I would like to understand what you did

Thank you for raising this point. We did not preserve the aspect ratio. We have amended the relevant section in methods to:

“For the OCT images, we resized each image to 224×224 pixels using bilinear interpolation and normalised each image individually based on its minimum and maximum pixel values to enhance local contrast and structural details.”

- sampling is mentioned to be stratified on gender, but was care taken to make sure both eyes (and all images) of a given patient are either in the training or test ? In other words, has sampling been performed at the patient level?

Sampling was performed at the patient level. All images belonging to one patient remained within its respective split with no data leakage. We have added a statement in methods to make clear “patient level split was performed to avoid data leakage”.

Finally regarding figure 2 and 4, it might be interesting to average results from the same sex and similar ages instead of random samples. Was this performed ?

Thank you for pointing this out. This is exactly what we did, hence we mentioned ‘averaged gradcam from 50 patients’. We will make this clearer in the methods section.

Reviewer: 2
Dr. Takefumi Yamaguchi, Tokyo Dental College
Comments to the Author:
This is an interesting study, reporting a novel DL model to predict biological sex and age using AS-OCT images. The manuscript is well-written and the study sounds fair.

Minor comments
1. I wonder if there were prediction error differences in ethnicity, age groups (younger than 30, or over 60) and disease (KCN, normal, and other disease) in the prediction, ROC-AUC, accuracy, and other performance indices.

Thank you for your comment. As stated in the manuscript under the subheading of “Subgroup Analysis”, “For age prediction, mean absolute errors were comparable across normal (5.3 years), keratoconus (5.3 years), and other pathologies (5.4 years), with no statistically significant differences between groups (p=0.28). Similarly, gender prediction showed consistent performance with ROC-AUC values of 0.74, 0.72, and 0.73 for normal, keratoconus, and abnormal groups respectively (p=0.42). Model performance was also evaluated across different ethnic groups. For age prediction, mean absolute errors revealed no statistically significant differences (p=0.39). For gender prediction, ROC-AUC values were compared across the same ethnic subgroups, also showing no statistically significant differences (p=0.26).”

The accuracy differences in age groups have been addressed in point 5, below

2. The authors included 557468 scans from 40592 eyes. Please describe how the authors selected the images.

As stated in the manuscript, “Data was extracted from all patients who underwent MS-39 imaging within our trust from October 2020 to March 2023. We opted not to exclude any images to reduce possible bias. The only filter we used were those suggested by the manufacturers (i.e. Placido >65% coverage or OCT section >85%).”

3. Please clarify "abnormal" eyes in detail.

Thank you for highlighting this. We classed abnormal as any cornea which was classified by the MS-39’s internal algorithm as ‘abnormal’ with a

probability score of >0.5. This likely referred to eyes which had significant corneal scarring or an ectasia other than keratoconus. This has been clarified in the methods section

4. I wonder if the accuracy of Placido exceeded AS-OCT or photos, because the tear film. Please address the comorbidity of dry eye disease.

Thank you for your comment, we feel that this is unlikely to be an explanation for the difference in accuracy as the quality of Placido images are typically more effected by dry eye than other forms of anterior segment imaging.

5. Page 15, Line 25: "There was also a clear trend towards less accurate age prediction for older subjects (Fig 3)." Please show this using some statistical analysis.

We have removed this line as no statistical analysis was performed on making this assertion and it will be computationally intensive to re-run model training at this late stage.
